# The untapped potential of ballistographic technology in behavioural sleep medicine

**DOI:** 10.1038/s41746-026-02350-w

**Published:** 2026-03-25

**Authors:** Yu-Hsuan Lin, Nicholas Meyer, Ta-Wei Guu

**Affiliations:** 1 Institute of Population Health Sciences, National Health Research Institutes, Miaoli County, Taiwan; 2 Department of Psychiatry, College of Medicine, National Taiwan University, Taipei, Taiwan; 3 Insomnia and Behavioural Sleep Medicine Clinic, University College London NHS Foundation Trust, London, UK; 4 Institute of Psychiatry, Psychology and Neuroscience, King’s College London, London, UK; 5 Division of Psychiatry, Department of Internal Medicine, China Medical University Beigang Hospital, Beigang, Taiwan; 6 School of Medicine, China Medical University, Taichung, Taiwan

**Keywords:** Business and industry, Computational biology and bioinformatics, Engineering, Health care, Mathematics and computing

## Abstract

Insomnia is the most prevalent sleep disorder globally, for which Cognitive Behavior Therapy for Insomnia (CBT-I) is the first-line treatment. We argue that under-mattress ballistography has the potential to enhance CBT-I delivery by providing treatment-relevant sleep metrics – sleep onset latency, wake after sleep onset, and sleep efficiency, with fewer limitations than polysomnography/actigraphy and consumer-wearable technologies. Validation studies show strong timing accuracy and acceptable sleep–wake classification. Integrating these metrics with CBT-I may improve decision-making, adherence, and outcomes. Future pragmatic trials and validation studies in psychiatric populations are needed.

## Introduction

Assessment and treatment of insomnia disorder are increasingly recognized as a key strategy for improving outcomes in psychiatric conditions, including mood, anxiety, and psychotic disorders, with which insomnia is highly comorbid^1^. Cognitive behavior therapy for insomnia (CBT-I), the recommended first-line insomnia treatment in most countries, currently relies on self-reported sleep diaries to track clinical progress and inform treatment. Although subjective measures will remain a cornerstone of insomnia treatment, augmenting these with metrics from objective tools promises to enhance the acceptability and effectiveness of CBT-I. Objective continuity metrics—particularly sleep onset latency (SOL), wake after sleep onset (WASO), and sleep efficiency—can complement diaries and provide a bias-resistant anchor for behavioral change during CBT-I.

## Limitations of current technologies

Existing sleep sensing technologies are of limited utility as adjuncts to CBT-I. The behavioral elements of CBT-I rely on five key parameters: SOL, WASO, time in bed (TIB), total sleep time (TST), and sleep efficiency (SE)^2^. Polysomnography (PSG) can be indispensable for ruling out other sleep disorders that may present with insomnia-like symptoms, such as obstructive sleep apnoea or periodic limb movement disorder—conditions particularly relevant in psychiatric populations where misdiagnosis is common. Many of the commonly reported PSG sleep architecture parameters, such as REM and sleep stages N1-N3, however, are not primary targets in behavioral sleep medicine. PSG is resource-intensive, not widely available, and poorly suited to long-term, multi-night monitoring in the home environment.

Actigraphy, on the other hand, offers practical advantages in outpatient settings by enabling longitudinal monitoring of rest–activity cycles, which can, for example, identify whether daytime inactivity or naps contribute to low homeostatic sleep drive, a common perpetuating factor in chronic insomnia. While more scalable than PSG, a major shortcoming of actigraphy is low specificity—a tendency to misclassify resting wakefulness as sleep. This is particularly problematic in insomnia, where individuals can spend prolonged periods lying awake in bed, trying to fall asleep.

A further limitation of current PSG and actigraphy technologies is their inability to directly measure “time in bed”, a critical denominator for calculating both SOL and sleep efficiency. For example, SOL is defined as the interval between lying down with the intention to sleep and actual sleep onset. While PSG can detect sleep onset through neurophysiological signals, it cannot capture the precise moment when an individual intends to fall asleep. Actigraphy, by contrast, estimates this intention indirectly, typically using ambient light data to infer the “lights-off” time—an approach that often fails to reflect the individual’s actual effort to initiate sleep. Consequently, estimates of SOL and efficiency still depend on participants’ self-reported “intended sleep time.” Adherence to sleep diaries is a common difficulty; however, with back-filled or biased entries posing a significant challenge^3^. Passive, bed-based monitoring can reduce documentation burden and provide a neutral shared reference when subjective estimates diverge from objective trends.

Most consumer wearable devices report only TST, without accurate estimates of SOL or SE. Yet meta-analyses of clinical trials of CBT-I have shown that TST is the least responsive insomnia parameter, with a small effect size of 0.16 (95% CI: 0.08–0.24). In contrast, SOL and WASO show moderate to large effect sizes of 0.57 (95% CI: 0.50–0.65) and 0.71 (95% CI: 0.61–0.82), respectively^4–6^. Consequently, even when patients experience meaningful clinical improvement, wearables may fail to detect it, depriving users of therapeutic feedback and potentially undermining their engagement with treatment. Moreover, many consumer devices emphasize unvalidated metrics such as “light sleep” and “deep sleep,” which are not treatment targets for insomnia and may mislead both patients and clinicians. For instance, in pharmacological treatment of insomnia, clinicians rarely base medication choices on the proportion of REM or non-REM sleep stages, or light or deep sleep. This divergence underscores the value of treatment-relevant continuity metrics—SOL, WASO, and SE—for tracking CBT-I progress.

## Ballistography

**Fig. 1:**
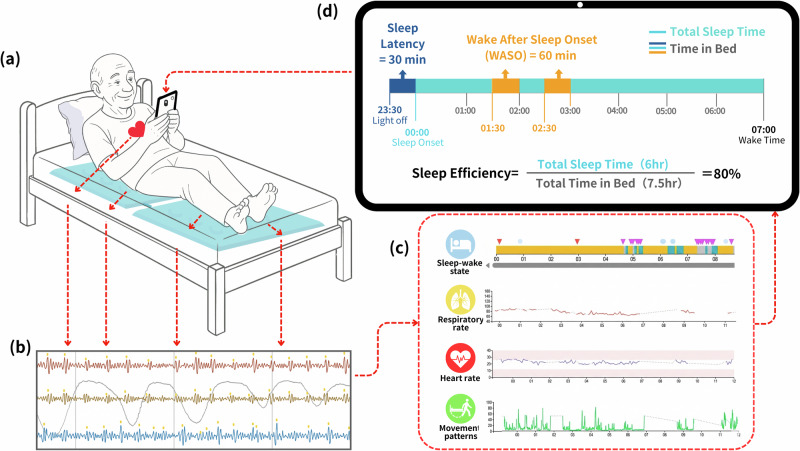
Contactless under-mattress ballistographic technology for sleep monitoring. **a** An under-mattress ballistographic sensor passively captures micro-vibrations related to cardiac ejection, respiration, and gross body motion without requiring the patient to wear a device. **b** The composite ballistocardiographic signal is processed to separate physiological components, including cardiac, respiratory, and movement-related activity. **c** These features are then used to derive time series of sleep–wake state, respiratory rate, heart rate, and movement patterns. **d** Continuous detection of bed presence provides a direct estimate of time in bed and supports calculation of insomnia-relevant continuity parameters. In the illustrated example, two wake periods after sleep onset (each 30 min) sum to 60 min of wake after sleep onset (WASO), with 6 h of total sleep time and 7.5 h of time in bed, corresponding to a sleep efficiency of 80%. Under-mattress ballistographic monitoring offers a non-invasive, low-burden method to track treatment response in cognitive behavior therapy for insomnia, particularly in psychiatric populations where maintaining accurate sleep diaries is challenging. Objective continuity trends can be reviewed alongside sleep diaries to guide stimulus-control and sleep-restriction dosing and to support shared decision-making when subjective reports diverge from objective patterns.

In this context, contactless sleep technology based on ballistographic signals offers a promising alternative Fig. 1. Ballistocardiography (BCG) classically refers to the measurement of ballistic forces generated by cardiac ejection, producing repetitive body motion with each heartbeat. Unlike PSG or actigraphy, under-mattress ballistographic systems can detect both bed presence and sleep–wake transitions without requiring physical contact or user interaction. Ballistocardiographic technology was originally developed to capture micro-movements generated by cardiac ejection forces during each heartbeat, a physiological phenomenon first demonstrated in the late 19th century. Clinical adaptation emerged in the 1940s with the development of direct-contact systems, which laid the groundwork for today’s non-contact sensing platforms that also capture respiratory activity and gross body movements in addition to cardiac signals^7^. In this Perspective, we therefore use the term “under-mattress ballistographic technology” as an umbrella for these BCG-based, multi-component mechanical signals used to estimate sleep continuity metrics relevant to CBT-I.

By enabling continuous and passive sensing of bed-presence and movement-defined transitions, the clinical value of under-mattress ballistography lies in the objective continuity endpoints it yields for CBT-I: (i) TIB; (ii) SOL, inferred from resting bed-presence preceding first sustained sleep; and (iii) WASO and SE derived from sleep–wake transitions. These metrics align with the behavioral components of CBT-I: stimulus control is associated with improvements in SOL and SE, whereas sleep restriction preferentially reduces WASO and also improves SE. By contrast, cognitive restructuring and third-wave approaches primarily influence subjective sleep quality rather than objective continuity^8^.

Recent validation studies support the performance of ballistography in real-world contexts. Large multi-night comparisons of under-mattress systems against PSG report strong agreement for key timing metrics (e.g., lights-out/bedtime, wake-up time) and acceptable sleep–wake classification for continuity metrics, while highlighting a tendency to overestimate TST when quiet wake is misclassified as sleep^9–13^. Community-based studies demonstrate that ballistography can track nocturnal sleep and daytime naps, supporting a 24-h view when used alongside actigraphy or diaries^10^. Smart-bed implementations of ballistography have further been validated for identifying sleep-disordered breathing^12^. Across recent evaluations, under-mattress ballistography reports epoch-by-epoch sleep–wake metrics (e.g., Cohen’s κ, area under the receiver operating characteristic curve (AUC), accuracy, sensitivity/specificity) and Bland–Altman bias with 95% limits of agreement for TST, SOL, and WASO. In community settings, contactless systems achieved ICCs > .75 for bed-on/bed-off timing but overestimated TST by ~93–101 min and sleep efficiency by ~9%; diary-free actigraphy also overestimated TST by ~47 min^10^. For sleep-disordered breathing screening at an apnoea–hypopnoea index (AHI) ≥ 15, a BCG smart-bed algorithm showed ~76% sensitivity and ~85% specificity versus PSG^12^. Collectively, these data indicate superiority of ballistography for bed-presence/timing (and in-bed nap detection), parity with actigraphy on overall accuracy, and ongoing constraints in wake specificity.

In routine care, combining objective data with sleep diaries could improve engagement and diary accuracy, providing a shared, low-burden record that outperforms passive tracking alone for clinical decision-making. Importantly, estimation of SOL remains an inference from continuous low-movement bed-presence, quiescence rather than a direct readout of intention to sleep; head-to-head studies using independent intention markers (e.g., synchronized annotations or button presses) are needed to quantify any advantage over light-based proxies.

Two practical limitations are inherent. Under-mattress systems require bed presence, meaning that unplanned daytime dozing or couch naps will be missed, and physical activity contributing to the sleep homeostatic process is not captured. When napping is reported, pairing under-mattress ballistography with actigraphy or wearables is recommended to capture out-of-bed sleep^10^ and the full rest-activity profile. A further practical challenge is that online media use in bed—an increasingly common activity—can generate extended periods of low-movement wakefulness, which may be misclassified as sleep and inflate continuity metrics unless supplemented by diary entries or contextual information. Future implementations may therefore need to incorporate contextual prompts or optional linkage to device-usage logs to better distinguish media-related immobility from true sleep.

Other contactless modalities warrant consideration. Radar-based approaches can infer hypnograms and respiratory events from radio-wave reflections^13^, and audio-based approaches estimate sleep from snoring and breathing acoustics via bedside devices or smartphones^14^. These are fully contactless but can be sensitive to room acoustics, occlusion, or placement. Ballistography, by contrast, directly senses mechanical physiology and continuous bed-occupancy. Radar may be preferable when access to the bed is restricted or when room-level respiratory sensing is prioritized, and audio may be useful when only a smartphone/bedside device is feasible but is less suitable in noisy or multi-occupant settings. Ballistography is advantageous when the clinical goal is precise bed-occupancy, sleep consolidation, and nightly continuity metrics. A goal-driven hybrid is therefore recommended: under-mattress ballistography for nocturnal continuity (latency/WASO/SE), actigraphy or wearables for 24-h behavior (including naps and activity), and radar or smart-bed solutions when respiratory screening is prioritized.

Despite their promise, several challenges remain before these technologies can be widely adopted in psychiatric practice. Accurate measurement of heart rate and respiration may require multiple synchronized sensors and complex real-time data integration using machine learning and cloud-based infrastructure. Moreover, most existing systems have not yet been rigorously validated in psychiatric populations. Unfamiliar user interfaces and proprietary data formats may further limit clinical uptake. Finally, cost remains a potential barrier to large-scale implementation, particularly in publicly funded healthcare settings. Crucially, objective tools should be framed as enablers of behavioral and relational work in CBT-I, and not as their replacements.

In the clinic, objective BCG parameters can enhance core behavioral strategies in CBT-I, without introducing additional burden. When ballistography shows prolonged SOL, clinicians can focus on stimulus control (clear contingencies for getting into and out of bed, leaving the bedroom when awake). When WASO predominates, therapists can calibrate the sleep-restriction window to consolidate sleep^8^. Passive under-mattress monitoring may reduce recall bias and back-filling, and provide an objective comparator when diary entries conflict with clinical impressions. Weekly or bi-weekly trend summaries of latency, WASO, and sleep efficiency displayed alongside brief diary aggregates can support shared decision-making, dose-adjustments to stimulus control or sleep restriction, medication reduction, and reinforcement of adherence. Approximately 30% of patients do not achieve a clinically significant response or remission after best-practice CBT-I^8^. For these patients, early identification of the core sleep problem (prolonged SOL vs. WASO) via passive, nightly monitoring can guide timely dose-adjustments and adherence troubleshooting.

Randomized controlled trials that study under-mattress ballistography as a feedback signal in CBT-I are currently lacking^12^. Further, external validity in psychiatric populations remains to be demonstrated in prospective studies that integrate objective continuity feedback into routine clinical practice. Beyond device validation, contemporary work shows that pairing CBT-I delivery with objective tracking is feasible and clinically promising—ranging from physician-assisted, internet-delivered CBT programs that integrate wearable-based monitoring in shift workers to pragmatic trials of prescription digital therapeutics delivering CBT-I in real-world settings^15,16^. These trajectories contextualize under-mattress ballistography as a low-burden source of continuity metrics (latency/WASO/SE) that can be embedded within digitally supported CBT-I platforms, and inform trials testing whether adding objective continuity feedback yields incremental benefit over standard care^15,16^.

Nevertheless, contactless sleep monitoring using ballistographic signals represents a meaningful step toward achieving objective, passive, and scalable assessment of insomnia in psychiatric care. Its alignment with the principles of behavioral sleep medicine and digital health makes it a compelling tool for supporting CBT-I. Future priorities include pragmatic trials of “CBT-I vs CBT-I + objective continuity feedback,” validation in psychiatric populations, user-centered interfaces integrated with routine workflows, and open, clinician-friendly data formats.

## Data Availability

Not applicable.
